# The Effect of Implicit Followership Antecedents on the New Generation of Individuals

**DOI:** 10.3389/fpsyg.2022.933770

**Published:** 2022-07-11

**Authors:** Kunxiu Lu, Wei Zhang, Xiaoyan Zhang, Junshu Feng, Ying Li

**Affiliations:** ^1^School of Management, Guangdong University of Science and Technology, Dongguan, China; ^2^School of Management, Northwest Normal University, Lanzhou, China; ^3^School of Economics and Management, Wuhan University, Wuhan, China

**Keywords:** core self-evaluation, proactive personality, implicit followership, positive implicit followership, negative implicit followership

## Abstract

This study used the Predisposition Proposition Theory of implicit followership to determine the effect of a proactive personality and core self-evaluation on the implicit followership of different schemas. Intertemporal survey data for one month from 452 university graduates were collected to evaluate that core self-evaluation significantly and positively affects positive implicit followership and significantly and negatively affects negative followership. However, the effect of proactive personality on implicit followership is not significant. The results of data analysis support the interpretation of propensity propositions in the study of personality traits. This study also determines the theoretical significance and practical application value.

## Introduction

Since the 1980s, with the spread of globalization, the economic environment has changed, especially in the United States, and the emergence of a technological revolution and intense competition has largely driven the transformation of corporate behavior patterns. Organizational structure tends to be a flat and rapid response to customer needs, cultivation of dynamic corporate adaptability, and the promotion of organizational change and innovation are important means to achieve a competitive advantage. The changing environment has given rise to a change in the perception of the role of followers by corporate managers: followers must become more proactive and must be an active part of the process of building competitive advantage within the company. However, within organizations, although leaders’ and managers’ perception of the follower role has shifted; it is more important to understand how followers themselves perceive the follower role and whether their traditional perceptions of the follower role have changed.

During the socialization process, members of organizations develop assumptions and schemas about the traits and behaviors that followers should demonstrate in their roles because of past work experience as followers, as defined by Implicit Followership Theories (IFTs) (Sy, 2010). IFT has two kinds of validity: positive implicit followership refers to an individual’s positive assumptions and schemas about the traits and behaviors that the follower role should have, which is also known as the followership prototype. Negative implicit followership refers to an individual’s negative assumptions and schemas about the traits and behaviors that the follower role should have, which is also known as the followership counter-prototype (Sy, 2010).

Implicit followership is espoused by individuals with different valences, but they are not two extremes of a continuous single dimension. Prototypes and counter-prototypes reflect different conceptions of implicit followership and are used to describe the representativeness and uniqueness of different implicit followership attributes (Sy, 2010). Followers’ implicit followership is stored in the follower’s brain and is activated when interacting with individuals in leadership positions. These followership schemas are the cognitive basis for organizational members to understand and respond to followership behaviors and are a necessary part of the “construction of meaning” in an organizational context (Weick, 1995).

The implicit schemas of Jelinek et al. (1983) allow individuals to develop a stable and potential structure of meaning over time, which affect their cognition, interpretive style, and behavioral style. In the context of implicit followership, implicitly perceived traits or behaviors do not represent the objective reality that is inherent to the individual: they represent perceptual abstractions and generalized labels that are used to classify followers and used as a basis for guiding followers in the traits and behaviors that they should exhibit.

The traditional view is that followers are submissive and obey leadership orders and arrangements (Baker, 2007). Previous studies determined how followers perceive their followership role and showed that different employees view the followership role in different ways. Some followers believe that as subordinates, they should remain silent, not participate in problem-solving or decision-making and support the leader’s way of doing matters (Carsten et al., 2010). Others believe that as a follower, they should collaborate with the leader to reinforce and support the leader’s agenda. This part of the individual watches followers as active co-producers or as participants with the leader in the leadership process (Carsten et al., 2010). Therefore, there are significant differences in the perceptions of the follower role by the followers themselves, which are both negative and proactive.

It is of interest to determine the reasons for the different perceptions of the follower role by followers and to determine the factors that lead to these differences in perceptions of follower roles in individuals in the same organizational context. Only a clear understanding of the reasons for these differences and a clear understanding of the different means of following that are exhibited by followers allows companies to guide and shape the perceptions of followers’ roles that are of high quality and beneficial to organizational development. This better promotes the healthy and sustainable development of the organization (Hoption and Han, 2021).

Few studies concern the antecedents of implicit followership. Some studies tentatively show that extroversion is one of the antecedent variables of implicit followership (Duong, 2011). There is interest in researching the origins of the follower hypothesis. It has been suggested that implicit theories develop through socialization experiences early in life (Epitropaki et al., 2013), such as interactions with parents (Keller, 2003). Goswami et al. (2017) showed that the personality traits of self-awareness, extraversion, and agreeableness that are displayed by followers predict the development of positive leader implicit followership. Some studies use attachment theory for antecedent research on implicit followership, but these studies determine the relationship between attachment style and implicit followership from a leader’s perspective, so leaders’ evasive and anxious attachment styles are negatively associated with their implicit followership prototypes and positively associated with their counter-prototypes (Thompson et al., 2018). Other studies demonstrate that the transformational parenting style of female primary caregivers is associated with their children’s prototype followership. There is also research that demonstrates a positive relationship between the transformative parenting style of female primary caregivers and their children’s positive implicit followings (Hoption and Han, 2021). As the internal and external environments of business organizations change rapidly and business managers perceive that followers must become more proactive, it is necessary to determine whether individuals with proactive personality traits are more likely to hold more positive perceptions of the follower role.

A proactive personality is a relatively stable tendency of individuals to influence environmental change. Individuals with a proactive personality are not constrained by environmental resistance and act proactively to change their external environment (Bateman and Crant, 1993). A proactive personality is a personality trait that features a willingness to change and is, perhaps, the most important personality trait in determining the success of individuals and organizations (Fuller and Marler, 2009). It has been demonstrated that a proactive personality is correlated differently with Big Five personality traits and that this trait contributes more to the prediction of certain variables than Big Five personality traits (Crant and Bateman, 2000).

Judge and Bono (2001) found that core self-evaluation is more important than Big Five personality traits. As an amalgamation of the four personality traits, core self-evaluation is more effective in explaining and predicting work outcomes (Judge and Bono, 2001), so two personality traits, proactive personality, and core self-evaluation, were used as antecedent variables for implicit followership for this study, which mentions that research on IFTs must involve an understanding of the origin of implicit followership schemas that are held by individuals.

The organization of this article is as follows. We review the relevant literature in the section “Background and Development of Hypotheses.” The section “Methodology” describes the development of research hypotheses to be assessed and the research methods used to generate the data set and test the model. The section “Results” presents the analysis results of empirical tests, and a discussion of these hypotheses and testing results follow thereafter. Finally, the section “Discussion” illustrates the conclusions from the findings, offers possible directions for future research, and discusses some limitations of this study.

## Background and Development of Hypotheses

### Proactive Personality and Followers’ Implicit Followership

Previous empirical research on proactive personality viewed a proactive personality as a positive individual trait and posited that individuals with high levels of proactive personality are more likely to perform better at work and to have positive job evaluations and good job outcomes (Chan, 2006). This study determines that proactive personality traits initially influence individuals’ perceptions of the follower role and then influence distal individual behavior.

Implicit followership is an individual’s perception of the follower role (Carsten et al., 2010; Sy, 2010) and includes both leaders’ implicit followership and followers’ implicit followership. Leaders’ implicit followership is the assumptions and schemas that leaders have about the behaviors and traits that followers should exhibit in their roles. Sy (2010) used factor analysis to classify implicit followership into positive and negative valence, using the principle of rationality. Positive implicit followership includes the traits of diligence, enthusiasm, and good citizenship; negative implicit followership includes the traits of incompetence, submissiveness, and disobedience.

This study concludes that high levels of proactive personality positively influence employees to develop cognitive schemas of positive implicit followership. A study of the correlation between a proactive personality and Big Five personality traits shows that a proactive personality is significantly and positively related to extroversion (Bateman and Crant, 1993), and that extroversion is one of the antecedent influences on implicit followership (Duong, 2011).

An analysis of recent literature on implicit followership theory shows that some studies propose dispositional propositions to explain the origin of implicit followership: individuals internalize and support specific IFTs over time, so they have a specific perception and view of the follower role (Engle and Lord, 1997; Sy, 2010). Duong (2011) showed that there is a significant connection between transformational leadership and leaders’ positive IFTs. Specifically, leaders’ positive IFTs play a mediating role between transformational leadership and extroversion. In terms of a followers’ implicit followership perspective, individuals with proactive personality traits tend to hold followers’ positive IFTs.

It has been shown that employees with proactive personalities positively perceive their role in the leadership process and hold beliefs about the co-creators of leadership (Torres, 2014). If employees hold more positive perceptual patterns about the characteristics and behaviors of their roles, they see themselves as partners with the leader or as co-creators of leadership (DeRue and Ashford, 2010). Individuals with high levels of proactive personality are also better at building interpersonal networks and they believe that extensive interpersonal relationships can help them improve their environment and their current situation (Fuller and Marler, 2009), so individuals with high levels of proactive personality are more inclined to help others and exhibit good citizenship behaviors.

It has been shown that a proactive personality and organizational citizenship behavior are significantly and positively correlated (Jong et al., 2015). Individuals who are not proactive do not exhibit organizational citizenship behavior: they do not recognize opportunities, do not seize opportunities to change things, and adapt passively to their environment and follow others, rather than change it. Therefore, the following hypothesis is proposed:

H1: Proactive personality has a positive influence on positive implicit followership.
H2: Proactive personality has a negative influence on negative implicit followership.

### Core Self-Evaluation and Followers’ Implicit Followership

An individual’s core self-evaluation is one of the crucial factors influencing employees’ intrinsic motivation (Shamir et al., 1993). The individual’s subjective assessment of the environment and others’ perceptions of the same environment in turn influence the individual’s evaluation of himself or herself (Sun et al., 2014). Core self-evaluation is the most basic evaluation of personal abilities and values (Judge et al., 1997). This evaluation stems from four personality trait dimensions: self-esteem, general self-efficacy, emotional stability, and locus of control.

Judge (2009) posits that core self-evaluation positively influences employees to form a cognitive schema of positive implicit followership because the definition of core self-evaluation states that core self-evaluation is the most basic evaluation and estimation of self-competence and value for an individual. If individuals have a high level of core self-evaluation, they have a higher opinion of their self-competence and value, so they value their significance in their job role more and perceive their job role more positively (Carsten et al., 2010).

Individuals with positive CSE have higher levels of task motivation, are more motivated, and can perform for a longer period (Erez and Judge, 2001). This success helps employees to develop positive work role perceptions during the work process. and traits such as self-efficacy, self-confidence, and optimism allow them to think, feel and act in ways that promote resource building and engagement in goal setting. In summary, individuals with high levels of positive CSE are committed to persistently pursuing their goals and are more competitive at work, so they more easily develop positive perceptions and schemas about their work roles.

This study argues that core self-evaluation negatively influences employees to form a cognitive schema of negative implicit followership. Employees with positive core self-evaluations are less susceptible to external environmental influences, are more likely to focus on the work itself, are more interested in the task itself, and more likely to enjoy the work process (Yoo and Lee, 2019), so this type of employee is less likely to engage in followership behaviors. Weiss (2002) argued that compared to subordinates with low self-esteem, subordinates with high self-esteem are less dependent on their work environment and more dependent on their self-perceptions, which in turn guide their task-related behaviors at work. This study concludes that high levels of self-esteem increase confidence in the ability to perform well in the work environment so they do not require environmental cues to demonstrate how they should perform. If an individual exhibits low levels of CES, the individual has lower self-competence and value assessments and is more likely to abandon their position and go with the flow if they feel they are incapable of doing something. Individuals perceive their value within the organization to be minimal.

A seven-year study by Headey and Wearing (1989) found that one of the traits of CSE neuroticism is negatively related to work events, such as unemployment and conflict with colleagues. Those who perceive themselves positively (e.g., high self-esteem and low neuroticism) also tend to pursue goals that are in harmony with themselves to a greater extent than people who perceive themselves negatively. On this basis, Judge and Larsen (2001) argued that positive individuals are more likely to evoke and pursue the achievement of work goals and negative individuals are more likely to avoid or prevent goal attainment because they fear a bad outcome. Therefore, individuals with low levels of negative CSE are more likely to avoid goal attainment, are less likely to have their own opinions, have a negative perception of themselves, and are more likely to conform to the opinions of others. Therefore, this study proposes the following hypothesis:

H3: Core self-evaluation has a positive influence on positive implicit followership.
H4: Core self-evaluation has a negative influence on negative implicit followership.

**FIGURE 1:**
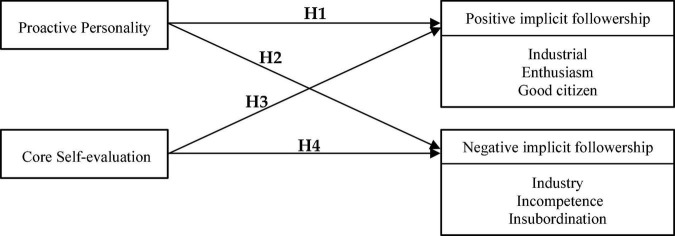
Theoretical framework.

Using the above four hypotheses, we proposed a research model for this study (as shown in Figure 1).

## Methodology

### Data Collection

The sample for the questionnaire survey was drawn from graduates of the School of Management in an undergraduate institution in the Guangdong region. To avoid homogenous errors, this study uses two-time points to collect questionnaires from this group of recently graduated students, with a one-month interval between the first and second surveys. The first questionnaire included questions about basic personal information, proactive personality, and core self-evaluation and the second questionnaire one month later included questions about positive and negative implicit followership.

Prior to the study, the Vice President of Teaching and Learning at the college’s School of Management was told the purpose and nature of the research. The questionnaire was then distributed to the leaders of the college and it was agreed that the questionnaire would be given to recently graduated students on the premise that the research does not invade the privacy of recently employed students and does not require confidential information about the companies in which the students were employed. The questionnaire was distributed as follows: firstly, the counselor of each major in the college was contacted and then the questionnaire was distributed to the employed students through Questionnaire Star. Students noted their school numbers in the questionnaire for post-matching purposes.

This study collected 658 fully completed first questionnaires and one month later used the same method to collect 611 questionnaires. All questionnaires that were filled in the same way were then eliminated and 452 valid samples were obtained after matching.

### Measurement Instrument

To ensure the validity of the content, the measurement items for this study are drawn from established scales. These scales were translated and backtranslated by postgraduate students in English and then the wording of the questionnaire was appropriately revised by two PhDs in Human Resource Management to ensure the accuracy of the translation. The sources of measurement items are:

• Proactive personality: This study uses a 10-item scale that was modified by Seibert et al. (1999) based on Bateman and Crant (1993). The scale is scored on a 7-point Likert scale from “totally disagree” to “totally agree”.
• Core self-evaluation: This dimension was adopted from the CSES scale that was developed by Judge et al. (2003), with 12 items. The scale is scored on a 5-point Likert scale from “totally disagree” to “totally agree”.
• Implicit followership: The scale was measured using the six dimensions of implicit followership that were developed by Sy (2010) to represent implicit followership with 18 questions, including “hardworking,” “enthusiastic,” and “good citizen,” three dimensions of nine questions indicating positive implicit followership, and “incompetence,” “conformity,” and “insubordination” three dimensions of nine questions indicating negative Implicit followership. Both validities were scored on a 7-point Likert scale from “totally disagree” to “totally agree.” The validation factor analysis resulted in the deletion of the items, “no work experience” and “soft-spoken” because factor loadings were less than 0.6. Therefore, the negative implicit followership construct has seven items.
• Control variables: To perform an accurate analysis of the research subjects and to exclude the interference from irrelevant variables to the questions of interest, the control variables included the relevant demographic variables of gender, age, and profession of the subjects.

## Results

### Descriptive Statistical Analysis

A total of 452 valid samples were obtained after matching this questionnaire. The number of male samples was 199 (44%) and the number of female samples was 253 (56%). There are more females than males. 345 subjects (76.3%) were aged 18–22, 106 (23.5%) were aged 23–26 and 1 (0.2%) was aged 27–30. All subjects are post-90s graduates. Employees in this age group will become the main force in enterprise development and will enable enterprises to gain a competitive advantage. About 164 subjects (36.3%) majored in marketing, 58 (12.8%) in logistics management, 229 (50.7%) in international trade and 1 (0.2%) in business administration. All 452 graduates had work experience (100%), so all graduates in this sample had found employment. Table 1 shows the sample structure for this study.

**TABLE 1:** Sample structure.

Category	Type	Frequency	Percentage
Gender	Male	199	44
	Female	253	56
Age	18–22	345	76.3
	23–26	106	23.5
	27–30	1	0.2
Major	Marketing	164	36.3
	Logistics Management	58	12.8
	International Trade	229	50.7
	Business Management	1	0.2
Availability of work experience	Yes	452	100
	No	0	0

Table 2 shows the values for mean, standard deviation, skewness, and kurtosis for each item. The lowest average score of all items is 1.92 for “uneducated” for the negative implicit followership construct. The highest average score is 5.89 for “team player” for the positive implicit followership. The lowest standard deviation is 0.892 for the items in the core self-evaluation construct. Skewness is between –1.591 and 1.575 and Kurtosis is between –0.662 and 3.866. Both sets of values meet Kline’s (2015) criteria for the skewness of less than 2 in absolute value for kurtosis of less than 7 in absolute value. These results exhibit univariate normality because they meet both criteria and the empirical data is suitable to use the structural equation model to evaluate the proposed model.

**TABLE 2:** Results of reliability and validity analysis.

Construct	Item	Mean	SD	Skewness	Kurtosis
Proactive Personality	PRO01	5.14	1.134	–0.401	0.321
	PRO02	5.33	1.182	–0.687	0.225
	PRO03	4.83	1.198	–0.051	–0.132
	PRO04	5.11	1.104	–0.399	0.395
	PRO05	5.23	1.224	–0.489	–0.067
	PRO06	4.88	1.120	–0.159	0.113
	PRO07	4.84	1.159	–0.065	–0.128
	PRO08	5.57	1.091	–1.010	1.498
	PRO09	4.80	1.219	–0.391	0.289
	PRO10	4.57	1.159	0.043	0.301
Core Self-evaluations	CSE01	3.80	0.993	–0.713	–0.066
	CSE02	2.69	0.928	0.631	0.397
	CSE03	3.35	0.904	–0.146	–0.208
	CSE04	3.04	1.019	0.186	–0.391
	CSE05	3.58	0.951	–0.476	–0.192
	CSE06	2.85	0.963	0.447	–0.249
	CSE07	3.36	0.952	–0.111	–0.540
	CSE08	3.38	0.892	–0.271	–0.248
	CSE09	3.83	1.002	–0.797	0.021
	CSE10	3.21	0.984	–0.020	–0.544
	CSE11	3.08	1.051	0.136	–0.662
	CSE12	3.46	0.918	–0.339	–0.280
Positive Implicit Followership	PIF 01	5.25	1.258	–0.720	1.056
	PIF 02	4.95	1.189	–0.593	0.963
	PIF 03	4.62	1.221	–0.156	0.674
	PIF 04	4.96	1.289	–0.477	0.414
	PIF 05	5.39	1.178	–0.683	1.161
	PIF 06	5.42	1.146	–0.611	0.885
	PIF 07	5.77	1.150	–1.177	2.293
	PIF 08	5.82	1.170	–1.439	3.172
	PIF 09	5.89	1.145	–1.591	3.866
Negative Implicit Followership	NIF 01	1.92	1.316	1.575	2.104
	NIF 02	2.14	1.320	1.019	0.473
	NIF 03	3.78	1.407	–0.246	–0.197
	NIF 04	3.23	1.369	–0.011	–0.509
	NIF 05	2.59	1.377	0.758	0.384
	NIF 06	2.43	1.357	0.898	0.515
	NIF07	2.56	1.377	0.697	0.044

### Reliability and Validity Analysis

Fornell and Larcker (1981) used three thresholds to measure convergent validity. The thresholds are for standardized factor loadings, composite reliability (CR), Cronbach’s Alpha, and analysis of the extracted mean variance extract (AVE). For each construct, the composite reliability implies the internal consistency of each indicator. Table 3 shows a summary of standardized factor loadings, composite reliability, and AVE. The standardized factor loadings for the items range from 0.670 to 0.926, so all scales are within the acceptable level of convergent validity.

**TABLE 3:** Results of reliability and validity analysis.

Construct	Item	Factor loading	CR	AVE	Cronbach’s alpha
Proactive Personality	PRO01	0.782	0.918	0.529	0.917
	PRO02	0.722			
	PRO03	0.768			
	PRO04	0.727			
	PRO05	0.700			
	PRO06	0.704			
	PRO07	0.738			
	PRO08	0.753			
	PRO09	0.670			
	PRO10	0.701			
Core Self-evaluations	CSE01	0.811	0.954	0.634	0.953
	CSE02	0.721			
	CSE03	0.818			
	CSE04	0.767			
	CSE05	0.793			
	CSE06	0.690			
	CSE07	0.804			
	CSE08	0.883			
	CSE09	0.820			
	CSE10	0.785			
	CSE11	0.756			
	CSE12	0.881			
Positive Implicit Followership	PIF 01	0.769	0.965	0.755	0.911
	PIF 02	0.915			
	PIF 03	0.857			
	PIF 04	0.702			
	PIF 05	0.911			
	PIF 06	0.882			
	PIF 07	0.917			
	PIF 08	0.911			
	PIF 09	0.926			
Negative Implicit Followership	NIF 01	0.746	0.936	0.676	0.857
	NIF 02	0.850			
	NIF 03	0.779			
	NIF 04	0.877			
	NIF 05	0.767			
	NIF 06	0.873			
	NIF07	0.853			

The CR value for latent variables ranges from 0.918 to 0.965, and Cronbach’s Alpha also ranges from 0.857 to 0.953, which exceeds the threshold of 0.6 that is recommended by Fornell and Larcker (1981), so all the constructs are internally consistent. The AVE values for each construct range from 0.529 to 0.755, which exceeds the value of 0.5 that is recommended by Hair et al. (2019) and Fornell and Larcker (1981). Therefore, the proposed model exhibits acceptable convergent validity.

This study compares the square root of the AVE for latent variables with the correlation between that for the latent variable and others, to calculate the discriminant validity (Fornell and Larcker, 1981). In Table 4, the bold numbers in the diagonal direction represent the square root of the AVE. All numbers in the diagonal direction are greater than the non-diagonal numbers, so discriminant validity is verified for all structures.

**TABLE 4:** Discriminant validity and correlations.

Construct	CSE	PRO	NIF	PIF
PRO	**0.796**			
CSE	0.050	**0.727**		
PIF	–0.310	–0.030	**0.876**	
NIF	0.290	0.090	–0.570	**0.869**

*PRO, Proactive Personality; CSE, core self-evaluation; PIF, positive implicit followership; NIF, negative implicit followership. The diagonal value is the square root of AVE.*

Analysis of the structural model for this study uses the maximum likelihood estimation method. Six common fit validation methods that were proposed in the Jackson et al. (2009) are used for this study’s goodness-of-fit metric. If the Chi-square value is divided by the degrees of freedom (DF), the ideal result is less than 3. There are more stringent criteria for model fit validation: the root mean square error of approximation (RMSEA) value must be less than 0.08 and the comparative fit index (CFI) must be greater than 0.9. The values for the indicators for this study are shown in Table 5.

**TABLE 5:** Model fit.

Model fit	Acceptance level	Model fit
Normed Chi-sqr (χ2/DF)	1 < χ^2^/DF < 3	1.172
RMSEA	<0.08	0.020
TLI (NNFI)	>0.9	0.990
CFI	>0.9	0.990
GFI	>0.9	0.940
AGFI	>0.9	0.930

*χ^2^, Chi-square; DF, degree of freedom; RMSEA, root mean square error of approximation; TLI (NNFI), Tucker-Lewis Index (Non-Normed Fit Index); CFI, comparative fit index; GFI, goodness of fit index; AGFI, adjusted goodness of fit index.*

### Structural Model

Table 6 shows the results for path coefficients to verify the causal relationship and calculate statistical significance. A proactive personality (β = 0.074, *p* > 0.05) does not have a significant effect on positive implicit followership, and a proactive personality (β = –0.014, *p* > 0.05) does not have a significant effect on negative implicit followership, so Hypotheses 1 and 2 are not supported. However, core self-evaluation shows a significant effect on positive implicit followership (β = 0.308, *p* < 0.01) and have a significant effect on negative implicit followership (β = –0.312, *p* < 0.01), so Hypotheses 3 and 4 are supported.

**TABLE 6:** Structural model result.

Endogenous Construct	Exogenous Construct	Unstandardized path coefficient	SE	Z-value	*p-value*	Standardized path coefficient (β)
PIF	PRO	0.061	0.043	1.397	n.s.	0.074
	CSE	0.280	0.051	5.504	***	0.308
NIF	PRO	–0.016	0.061	–0.260	n.s.	–0.014
	CSE	–0.391	0.070	–5.588	***	–0.312

*PRO, proactive personality; CSE, core self-evaluation; PIF, positive implicit followership; NIF, negative implicit followership.*

***p < 0.001; n.s., non-statistical significance.

## Discussion

### Discussion

This study determines the effect of different personality traits on individual positive and negative implicit followership using data from a sample of 452 graduates who graduated in three majors from the management department of an undergraduate college in southern China. The results show that employees with a higher core self-evaluation are more likely to form positive implicit followership perceptions.

Using the principle of the “dispositional proposition” of implicit followership, this study determines the effect of a proactive personality and core self-evaluation for employees on implicit followership. A proactive personality tends to make intentional changes to the environment (Bateman and Crant, 1993) and core self-evaluation represents the individual’s basic self-assessment of self-worth, competence, and strengths (Judge et al., 2005).

The “will do” motivation of a proactive personality and the “can do” attitude of core self-evaluation influence the extent to which individuals participate in work roles (Haynie et al., 2017). Individuals with highly proactive personalities use their involvement in work to build structure and social resources (Bakker et al., 2012), but individuals with high core self-evaluations engage in work with a coping style to solve problems, rather than using effort to avoid coping (Kammeyer-Mueller et al., 2011). Therefore, there is a difference in the focus and purpose of the two different personality traits in relation to work engagement.

The results of the study show that a proactive personality does not have a significant effect on either positive or negative implicit followership, but core self-evaluation has a significant positive effect on positive implicit followership and a significant negative effect on negative implicit followership. Besides, the results of this study show that a proactive personality does not have a significant effect on either positive or/negative implicit followership, possibly because implicit followership is a perception of a role, rather than an actual employee behavior, so individuals with a proactive personality are more concerned with the possible effects and changes that result from the consequences of their implementation of proactive behavior.

A proactive personality tends to change environmental conditions, with the ultimate aim of identifying opportunities to act until meaningful change occurs (Crant and Bateman, 2000). Some studies show that whether the relationship between a proactive personality and employee outcomes is positively correlated depends largely on the boundary conditions. Chan (2006) showed that a proactive personality is a significant positive predictor of supervisory support if an individual’s situational judgment is very effective, but if an individual’s situational judgment is not effective, a prospective personality is a significant negative predictor of the latter.

Sun and van Emmerik (2015) also found that if individuals possess few political skills, a proactive personality is negatively correlated with supervisory evaluations, but this negative correlation does not apply if the individual has good political skills. Therefore, the results for the effect of a proactive personality are not similar to those for core self-evaluation of more positive and challenging coping aspects of an individual’s work (Tims and Akkermans, 2017).

### Theoretical Contributions

This study makes the following theoretical implications. It contributes to the literature on the antecedents of implicit followership research by determining the links between a proactive personality, core self-evaluation, and implicit followership. In a review of research on implicit leadership and implicit followership theory, Epitropaki et al. (2013) noted that research on IFTs must first determine the origin of implicit followership schemas that are held by individuals and the antecedent influences on implicit followership and suggested that future studies should explore a broader range of personality traits to determine the antecedent variables of implicit followership. Lord et al. (2020) also noted the possibility that personality traits and core self-evaluation may be antecedent influences on either implicit leadership or implicit followership, in response to the currently limited research on implicit leadership and implicit followership. This study shows that core self-evaluation is significantly and positively related to positive implicit followership.

This study contributes to positive psychology literature. Positive implicit followership is a positive belief that an individual has about the role of a follower. If individuals exhibit positive implicit followership, positive cognitive structures enhance beliefs and confidence in influencing an organization, so they believe that they can achieve their full potential through self-fulfilling prophecies (Torres, 2014). This shows how personality traits are associated with individuals shaping positive implicit followings.

This study contributes to research on employee-centered followership. Most studies of implicit followership show that individual implicit followership perceptions play an important role in the formation of employee followership, but these studies are mostly based on the influence of implicit followership and there is a lack of empirical research on the influence of antecedents of employees’ implicit role perceptions from the employees’ perspective and at the individual level. This study determines employees’ antecedents of implicit followership and analyzes the results of the influence of a proactive personality and core self-evaluation on implicit followership. The results show that individuals with high assessments of self-competence and value are more likely to develop positive implicit followership perceptions, which contribute to the development of the self.

### Practical Implications

Several practical implications are proposed as follows. First, this study determines the causal correlation between a proactive personality and core self-evaluation and implicit followership. This study has the following practical implications. The study applies to the recruitment and selection of talent in companies. Studies show that core self-ratings significantly predict individuals’ perceptions of positive implicit followership, so companies must identify employees with high core self-ratings during the selection process.

Second, a proactive personality does not significantly predict implicit followership and studies show that proactive personality traits are a double-edged sword and that a proactive personality and core self-evaluation are two personality traits with different predictive validity. Therefore, when recruiting talent, companies can use a proactive personality as a secondary personality for talent selection and use a multi-dimensional approach for recruiting suitable talent.

Third, companies must invest in design and training. Core self-evaluation is a personality trait with four personality trait dimensions: self-esteem, general self-efficacy, emotional stability, and locus of control. Some studies show that self-efficacy is an individual state that can change, so core self-evaluation is malleable. Therefore, managers, including those from human resources and the employee’s immediate supervisors, can help employees to improve their self-efficacy by intervention. They can encourage and motivate employees with words to boost confidence and provide training that better suits employees’ knowledge, skills, and abilities, so that employees feel that their self-efficacy has improved. This can increase core self-evaluation levels and make it easier for them to shape their perceptions of positive implicit followership.

### Research Limitations and Future Works

This study has some shortcomings and deficiencies due to various limitations. First, the sample for this study consists of graduates from undergraduate colleges and there are differences in the perceptions of the role of followers between school graduates and the new generation of employees entering the company. This study is also conducted in a region that was the first to be reformed and where the economy is more developed than in the rest of China. In this region, perceptions were influenced by Western ideas earlier than in other parts of China.

Second, this study does not determine whether there are boundary conditions for the process of the influence of personality traits on implicit followership. Since the beginning of China’s reform, Western thought and culture have permeated into China and traditional Chinese culture and Western modern culture are contradictory and intertwined. Individuals with high power distance are more likely to obey authority and leaders so they may be more likely to develop negative implicit followership cognitive schemas. Paternalistic and authoritarian leadership styles in the Chinese context are also a subject for future study.

Finally, this study found uses empirical analysis to show that proactive personality traits do not have a significant effect on individuals. Previous studies show that the results for a proactive personality are significantly related to boundary conditions and the current impact on a proactive personality is more supportive of its positive results. There are few negative findings for proactive personality traits so future studies might determine the effect of this personality trait and the effect of boundary conditions.

## Data Availability Statement

The raw data supporting the conclusions of this article will be made available by the authors, without undue reservation.

## Ethics Statement

Ethical review and approval was not required for the study on human participants in accordance with the local legislation and institutional requirements. Written informed consent from the patients/participants or patients/participants legal guardian/next of kin was not required to participate in this study in accordance with the national legislation and the institutional requirements.

## Author Contributions

KL, WZ, and XZ: conceptualization and methodology. KL, WZ, JF, and YL: formal analysis, investigation, and visualization. KL, WZ, XZ, JF, and YL: writing about original draft preparation. KL, WZ, XZ, and YL: writing about review and editing. All authors have read and agreed to the published version of the manuscript.

## Conflict of Interest

The authors declare that the research was conducted in the absence of any commercial or financial relationships that could be construed as a potential conflict of interest.

## Publisher’s Note

All claims expressed in this article are solely those of the authors and do not necessarily represent those of their affiliated organizations, or those of the publisher, the editors and the reviewers. Any product that may be evaluated in this article, or claim that may be made by its manufacturer, is not guaranteed or endorsed by the publisher.
